# Possible association between oral health and sleep duration: A cross-sectional study based on the Korean National Health and Nutrition Examination Surveys from 2010 to 2015

**DOI:** 10.1097/MD.0000000000028035

**Published:** 2021-12-03

**Authors:** Sungjun Han, Donghyun Jee, Yun-Jin Kang, Yong-Jin Park, Jung-Hae Cho

**Affiliations:** a Department of Otolaryngology-Head and Neck Surgery, College of Medicine, The Catholic University of Korea, Seoul, Korea; b Department of Ophthalmology, College of Medicine, The Catholic University of Korea, Seoul, Korea.

**Keywords:** Oral health, sleep duration, risk factor

## Abstract

This study was performed to investigate the association between oral health and sleep duration in South Korean subjects using 2010–2015 data from the Korean National Health and Nutrition Examination Survey (KNHANES).

Cross-sectional data on 35,599 adults over the age of 19 years who completed KNHANES were analyzed. All participants reported subjective oral health status and their daily average sleep duration using a self-reported questionnaire. Sleep duration and oral health status were divided into 3 categories: ≤5, 6–8, ≥9 h/day and good, fair, poor, respectively.

The overall prevalence of poor oral health status was 43.8%. Univariate analysis demonstrated that poor oral health status was significantly associated with age, smoking, alcohol, diabetes, education, income, depression, marital status, and sleep duration. After adjusting for covariates (age, sex, diabetes mellitus, hypertension, obesity, smoking, income, education, marital status), sleep durations of ≤5 hours (OR = 1.42; 95% CI, 1.26–1.60) and ≥9 hours (OR = 1.21; 95% CI, 1.04–1.40) were significantly associated with poor oral health, compared to a sleep duration of 6–8 hours. Short or long sleep duration was more likely to have an impact on the development of poor oral health status in men than in women. A significant relationship between sleep duration and oral health status was found in participants younger than 60 years.

This is the first report that both short and long sleep durations are significantly associated with the development of poor oral health status. The effect of short or long sleep duration on poor oral health was more significant in younger subjects and in men.

## 1 Introduction

Oral health has been regarded as a mirror of the general health.^[1]^ Many studies have shown that oral health status is related to diseases of various organs (respiratory, cardiovascular, gastrointestinal, neurological, and endocrine disorders, such as diabetes mellitus).^[2–6]^ Oral health includes the ability to speak, smile, taste, touch, chew, and swallow foods. The World Health Organization defines oral health as “being free of chronic oro-facial pain, oral and pharyngeal (throat) cancer, oral tissue lesions, birth defects such as cleft lip and palate, and other diseases and disorders that affect the oral, dental and craniofacial tissues, collectively known as the craniofacial complex.”^[7]^ A healthy mouth enables not only nutrition of the physical body but also enhances social interaction and promotes self-esteem and feelings of well-being.

Poor oral health status can be a manifestation of disease from other organs and have a significant influence on health-related quality of life.^[8–10]^ Many studies have demonstrated relationships between oral health and other health factors, including quality of life, depression, metabolic syndrome, and mortality.^[4,5,11–13]^

Appropriate sleep duration is another important factor of an individual's quality of life and general health. Short or long sleep duration is also associated with various diseases, cardiovascular disease, cerebrovascular accidents, obesity, cancer, and even mortality.^[14–18]^ Based on previous studies, we hypothesized that inappropriate sleep duration may be associated with poor oral health status. However, no population-based studies have yet examined the relationship between oral health status and sleep duration in adults. Therefore, this large, national population-based study was performed to investigate the relationship between oral health and sleep duration.

## 2 Materials and methods

### 2.1 Ethical consideration

Written informed consent was obtained from all participants prior to the survey. And approval for this study was obtained from the Institutional Review Board of The Catholic University of Korea in Seoul, South Korea (IRB No. VC18ZESI0236).

### 2.2 Study population and design

**Figure 1:**
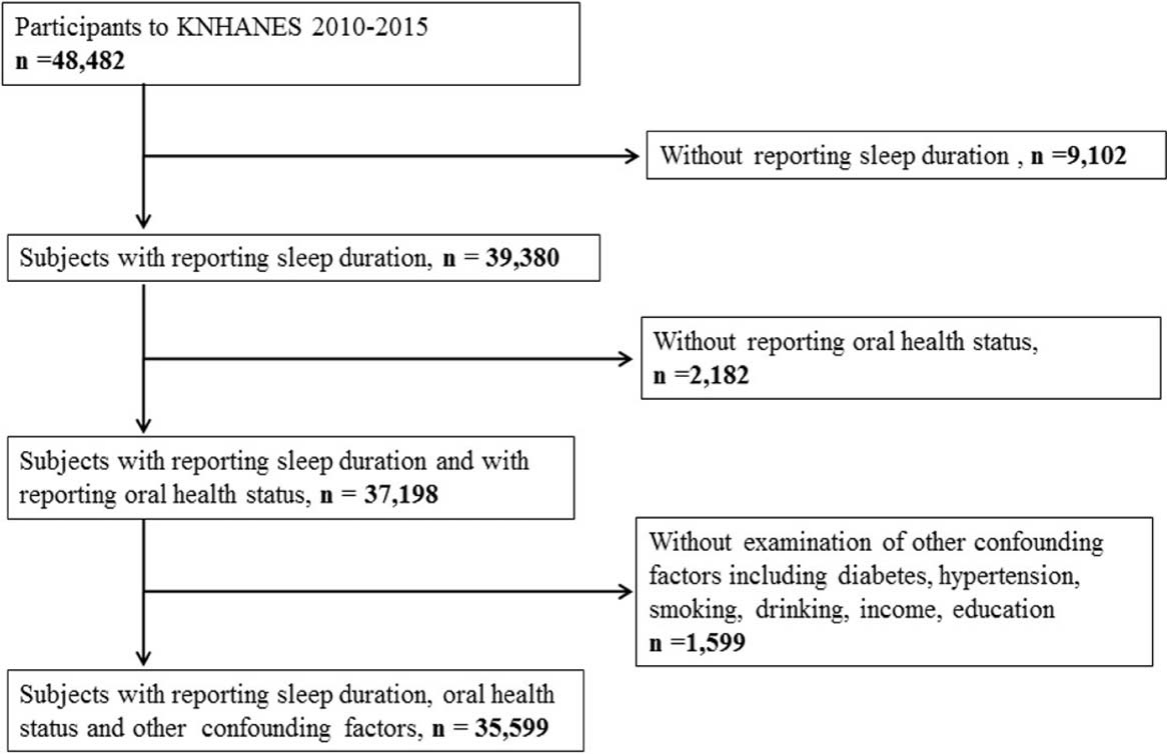
Flow diagram presenting the selection of study participants. A total 35,599 subjects were included in the analysis.

This study was a cross-sectional analysis based on data from a nationwide health survey of the fifth (2010–2012) and sixth (2013–2015) editions of the Korea National Health and Nutrition Examination Survey (KNHANES) for 6 years combined. KNHANES is a government-sponsored survey conducted by the Korean Center for Disease Control and Prevention, which commenced in 1998. The participants of KNHANES are a nationally representative sample of the Korean population. The KNHANES data include health interviews, health examinations, nutrition surveys, and laboratory investigations. The total number of participants in KNHANES (2010–2015) came to 48,482. Here, we excluded participants without reports of oral health status (n *=* 2182) or sleep duration (n = 9,102). Participants without reports of diabetes mellitus, hypertension, smoking, alcohol drinking, income, and education were also excluded (n = 1599). Therefore, 35,599 subjects were included in the analysis (Fig. 1).

### 2.3 Demographic variables

Demographic data and information on health-related behaviors were collected by self-reported questionnaires and during personal interviews. The demographic and socioeconomic variables in the survey included gender, age, household income, education, and marital status. The health behavior variables included smoking, alcohol drinking, and the presence of diabetes mellitus or hypertension. Subjects were divided into 3 groups according to smoking history based on self-reported cigarette usage:

1. current smoker, those who smoke currently and had smoked ≥100 cigarettes;
2. past-smoker, those who had smoked in the past but had stopped smoking for more than 12 months; and
3. nonsmoker, those who had never smoked or had smoked <100 cigarettes in their lifetime.

Subject who drank more than 30 g/day of alcohol was designated as drinkers. Body mass index (BMI) was calculated as BMI = weight (kg)/height (m)^2^. Obesity was defined as BMI > 25 kg/m^2^. A diagnosis of diabetes was assigned to subjects who reported a history of diagnosis by a physician, those who were receiving treatment with drugs for diabetes, such as insulin or oral hypoglycemic agents, or those with a fasting plasma glucose level >126 mg/dL. The presence of hypertension was defined as systolic blood pressure ≥140 mm Hg, diastolic blood pressure ≥90 mm Hg, or use of antihypertensive medication. Blood pressure was measured with a sphygmomanometer with the patient in the seated position. After collecting 3 measurements at 5-minute intervals, the average of the second and third measurements was used for the analysis.

The mental health variables were self-perceived levels of stress, depression, and suicidal ideation. The variables of self-perceived level of stress, depression, and suicidal ideation were classified according to “yes” or “no” responses to the questions, “How stressed are you on a daily basis?,” “Have you experienced a continuous feeling of sadness or despair for over 2 weeks that interfered with your daily activities in the last year?,” and “Have you considered committing suicide in the last year?”

### 2.4 Survey of oral health status and sleep duration

Participants ≥19 years were asked about subjective oral health status and the oral status was examined by trained dentists. Self-reported oral health status was classified according to a 5-point Likert scale depending on the response to the question, “How do you feel about your oral health related to the teeth, gums, tongue, etc.?” as follows: 5, very good; 4, good; 3, fair; 2, poor; or 1, very poor.

Sleep duration was self-reported. All participants were asked: “How many hours do you usually sleep (1–24 hours) every night?”

### 2.5 Statistical analysis

Statistical analyses were performed using SPSS software version 18.0 (SPSS, Inc., Chicago, IL). Strata, sampling units, and sampling weights were used to obtain point estimates and standard errors. The demographic characteristics were described using means and standard errors for continuous variables and percentages and standard errors for categorical variables. We compared the demographic characteristics using the Chi-Squared test. To evaluate the association between sleep duration and oral health status, 5-point scaled self-ratings of oral health was re-classified into 3 groups: good (very good or good); fair; and bad (poor or very poor). In addition, sleep duration was categorized into 3 groups: ≤5, 6–8, and ≥9 hours per day.^[19,20]^

Univariate and multivariate logistic regression analyses were performed. After calculating the crude odds ratios (OR), they were adjusted for age, sex, and other factors, including diabetes mellitus, hypertension, obesity, smoking, income, education level, and marital status. Participants were stratified by gender to examine how the risk factors for oral status differ between men and women. For trend analysis, we evaluated changes in ORs according to long and short sleep duration based on normal sleep (6–8 hours). We examined all variables in the logistic regression analysis for multicollinearity, and only variables with a variance inflation factor <5 were used. All *P*-values were 2-tailed, and *P* < .05 was taken to indicate statistical significance.

## 3 Results

**Figure 2:**
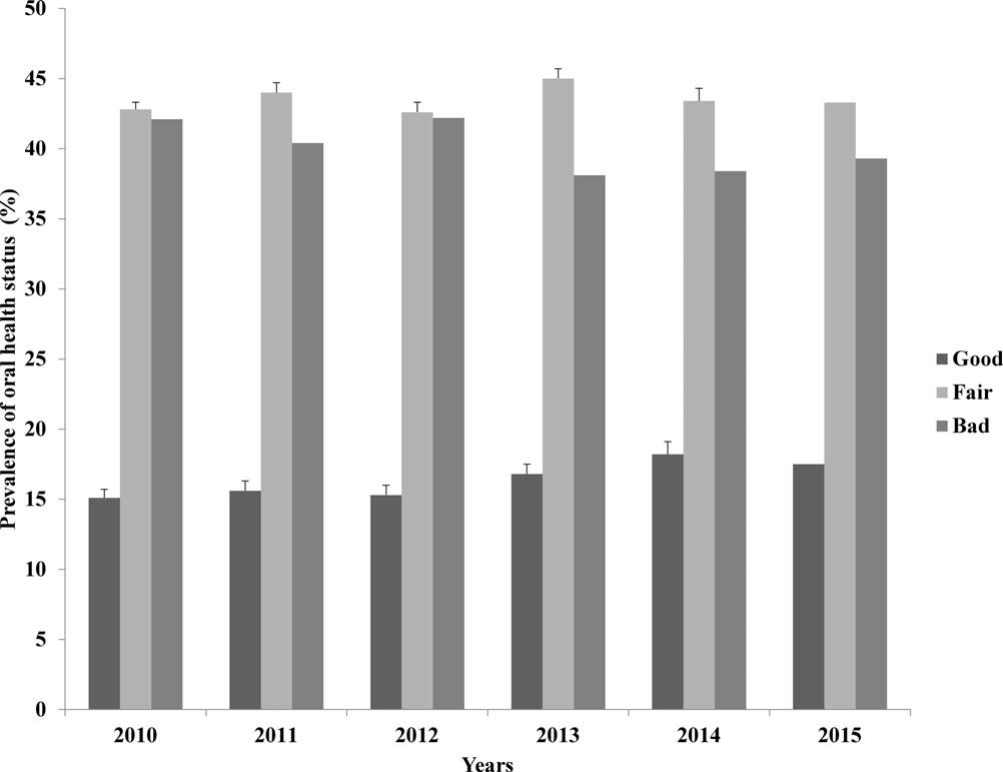
Changes in oral health status over 6 years (2010–2015). The distribution of subjective oral health status categorized by 3 groups (good, fair, and bad) is shown.

**Figure 3:**
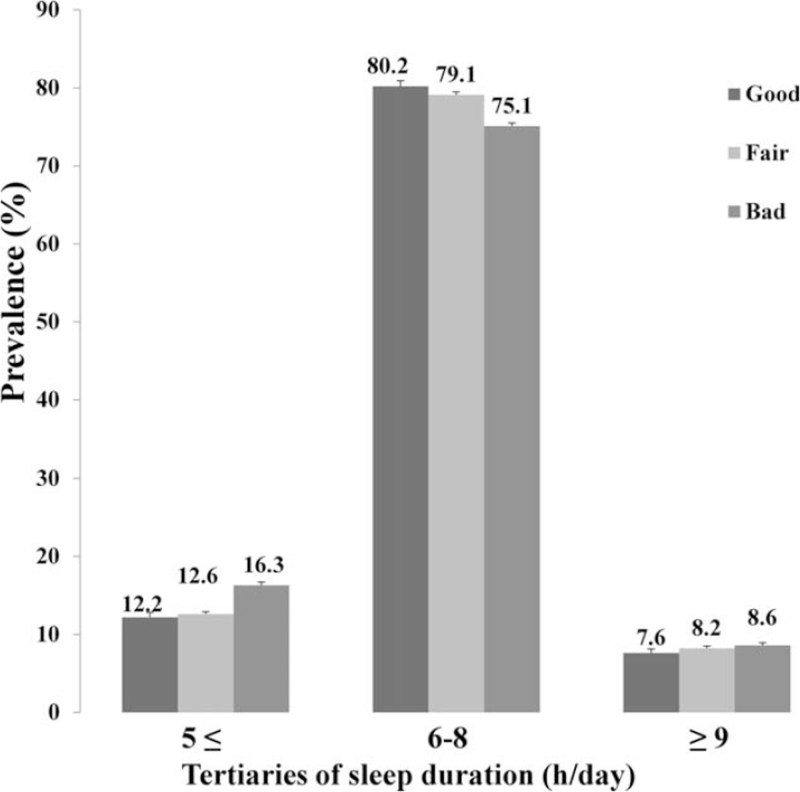
Prevalence (%) of 3 groups of oral health status according to sleep duration (≤5, 6–8, and ≥9 hours per day).

The overall prevalence of poor oral health status was 43.8%. The distribution of subjective oral health status over the 6-year study period is shown in Figure 2. The overall oral health status during the study period improved gradually, with the ratio of poor oral health status decreasing to 42.1% in 2010 and to 39.3% in 2015. The demographic and clinical characteristics according to the oral health status are shown in Table 1. Poor oral health status was significantly associated with female sex, older age, alcohol consumption, smoking status, diabetes mellitus, lower income, educational level, marriage, mental stress, depression, and short or long sleep duration (*P* < .05). Figure 3 shows that the prevalence of poor oral health status was higher in groups with short or long sleep duration (*P* < .001). In particular, we found that short sleeping time had a greater impact on the development of poor oral health than long sleeping time.

**Table 1:** Demographic and clinical characteristics according to oral health status, as reported in the Korean National Health and Nutrition Examination Survey 2010–2015.

Characteristics	Good (n = 5086)	Fair (n = 14,920)	Poor (n = 15,593)	*P*	Participants (n = 35,599)
Male (%)	53.1 (0.7)	48.0 (0.4)	50.8 (0.5)	<.001^∗^	50.0 (0.2)
Age (yrs)	33.8 (0.3)	35.6 (0.2)	44.4 (0.2)	<.001^∗^	38.9 (0.1)
Obesity (%)	32.1 (0.8)	30.5 (0.5)	34.2 (0.5)	.110	32.4 (0.3)
Alcohol drinker (%)	81.8 (0.6)	82.9 (0.4)	86.1 (0.3)	<.001^∗^	84.1 (0.3)
Smoking status				<.001^∗^	
Current (%)	12.3 (0.5)	15.8 (0.4)	26.8 (0.5)		19.6 (0.3)
Former (%)	15.3 (0.5)	14.8 (0.3)	17.5 (0.3)		16.0 (0.2)
Never (%)	72.4 (0.7)	69.4 (0.4)	55.7 (0.5)		64.4 (0.3)
Diabetes (%)	7.8 (0.5)	6.7 (0.3)	11.0 (0.3)	<.001^∗^	8.8 (0.2)
Hypertension (%)	26.1 (0.8)	22.9 (0.5)	29.8 (0.5)	.958	26.4 (0.4)
Income (quartile)				<.001^∗^	
1st (lowest)	23.6 (0.8)	24.7 (0.6)	28.9 (0.6)		26.2 (0.5)
2nd	24.0 (0.7)	25.3 (0.5)	26.6 (0.5)		25.6 (0.4)
3^rd^	26.2 (0.7)	25.0 (0.5)	23.7 (0.5)		24.7 (0.4)
4th (highest)	26.2 (0.8)	25.0 (0.6)	20.8 (0.5)		23.5 (0.5)
Education				<.001^∗^	
Elementary	40.6 (0.7)	29.6 (0.4)	26.3 (0.5)		30.1 (0.3)
Middle school	9.3 (0.4)	11.6 (0.3)	12.8 (0.3)		11.7 (0.2)
High school	25.3 (0.7)	30.0 (0.5)	34.9 (0.5)		31.2 (0.4)
University	24.8 (0.7)	28.7 (0.5)	25.9 (0.6)		27.0 (0.4)
Marital status_married (%)	51.0 (0.7)	56.8 (0.5)	73.6 (0.5)	<.001^∗^	62.6 (0.3)
Mental stress_experienced	21.3 (0.7)	24.6 (0.4)	31.0 (0.5)	<.001^∗^	26.9 (0.3)
Depression_experienced	8.5 (0.5)	10.6 (0.3)	15.3 (0.4)	<.001^∗^	12.4 (0.4)
Suicide feeling_experienced	6.9 (0.5)	9.0 (0.3)	13.0 (0.4)	.043^∗^	10.5 (0.2)
Sleep duration (h/day, %)				<.001^∗^	
≤5 h	12.2 (0.6)	12.6 (0.3)	16.3 (0.4)		14.1 (0.2)
6–8 h	80.2 (0.7)	79.1 (0.4)	75.1 (0.4)		77.6 (0.3)
≥9 h	7.6 (0.5)	8.2 (0.3)	8.6 (0.3)		8.3 (0.2)

Odds ratios and 95% confidence intervals (95% CIs) were obtained by multivariable logistic regression analysis. The risks of poor oral health status in the 3 subgroups categorized by sleep duration were calculated. Table 2 shows that poor oral status was significantly associated with sleep duration. After adjusting for covariates (sex, age, diabetes, hypertension, obesity, smoking, income, education, and marriage), a sleep duration of ≤5 hours (OR = 1.42; 95% CI, 1.26–1.60) and a sleep duration of ≥9 hours (OR = 1.21; 95% CI, 1.04–1.40) were significantly associated with the development of poor oral health status, compared to a normal sleep duration of 6–8 hours. An association between poor oral health status and short or long sleep duration was seen in men after adjusting for variables. In men, a sleep duration of ≤5 hours (OR = 1.26; 95% CI, 1.02–1.58) and a sleep duration of ≥9 hours (OR = 1.34; 95% CI, 1.02–1.75) were significantly associated with poor oral health, compared to a normal sleep duration of 6–8 hours. For women, only short sleep duration (OR = 1.28; 95% CI, 1.09–1.52) was associated with poor oral health compared to a normal sleep duration of 6–8 hours. We analyzed the relations of oral health status and sleep duration according to age. For the younger age group (<60 years old), a sleep duration of ≤5 hours (OR = 1.41; 95% CI, 1.17–1.70) and a sleep duration of ≥9 hours (OR = 1.27; 95% CI, 1.02–1.58) were significantly associated with poor oral health, compared to a normal sleep duration of 6–8 hours. However, no association between poor oral health status and short or long sleep duration was seen in participants more than 60 years old.

**Table 2:** Adjusted odds ratios (95% confidence intervals) for the prevalence of poor oral health status according to sleep duration.

Sleep duration (h/day)	≤5 h	6–8 h	≥9 h
Total			
Good	1.00	1.00	1.00
Fair	1.05 (0.93–1.18)	1.00	1.09 (0.94–1.27)
Poor	*1.42 (1.26–1.60)* ^∗^	1.00	*1.21 (1.04–1.40)* ^∗^
By gender			
Males			
Good	1.00	1.00	1.00
Fair	1.03 (0.83–1.29)	1.00	*1.26 (1.01–1.58)* ^∗^
Poor	*1.26 (1.02–1.58)* ^∗^	1.00	*1.34 (1.02–1.75)* ^∗^
Females			
Good	1.00	1.00	1.00
Fair	*1.18 (1.01–1.39)* ^∗^	1.00	1.02 (0.81–1.28)
Poor	*1.28 (1.09–1.52)* ^∗^	1.00	1.02 (0.82–1.26)
By age			
Age < 60 years old			
Good	1.00	1.00	1.00
Fair	1.13 (0.94–1.36)	1.00	1.08 (0.86–1.34)
Poor	*1.41 (1.17–1.70)* ^∗^	1.00	*1.27 (1.02–1.58)* ^∗^
Age ≥ 60 years old			
Good	1.00	1.00	1.00
Fair	1.16 (0.97–1.40)	1.00	1.11 (0.84–1.46)
Poor	1.12 (0.93–1.34)	1.00	1.11 (0.86–1.42)

## 4 Discussion

A U-shaped association has been reported between sleep duration and mortality.^[18]^ In this study, the association between sleep duration and oral health showed a similar type of graph as a V-shaped curve. It meant that short or long sleep duration was associated with poor oral health.

Poor oral health has been shown to be strongly associated with mortality.^[11,21]^ Known risk factors for poor oral health include smoking, alcohol, diet, metabolic disorders, low socioeconomic status, and mental health.^[13,22–26]^ In this study, we also found that poor oral health status was significantly associated with older age, smoking, alcohol consumption, diabetes mellitus, education level, income level, degree of mental stress, depression, marital status, and short or long sleep duration. Alcohol dependence has been reported as a possible risk factor for periodontal disease, which is an important component of oral health.^[24]^ Periodontal problems in alcohol-dependent subjects were primarily associated with poor oral hygiene and poor dental care. Another cross-sectional study has also evaluated the effects of alcohol consumption on the severity of periodontal disease.^[27]^ Beneficial effects of marriage on cancer outcomes have been observed in oral cavity and laryngeal cancers.^[28]^ In contrast, marital status had a negative impact on oral health status in the present study. That is, marital status, such as divorce and never married, had a positive impact on oral health status. The recent meta-analysis has shown a similar result that married participants were 1.30 times more likely to have poor oral health related quality of life.^[29]^ Pearson et al also reported that marital status had a limited impact on periodontal health but may have a great impact on systemic conditions in people who are divorced or have never married.^[30]^

Periodontal diseases are closely related with oral health status. It has been reported that sleep duration is positively correlated with the prevalence of periodontitis, which increased the estimated odds of periodontitis by 1 more hour of sleep compared to a sleep duration of 5 hours.^[31]^ According to the results of this study, longer sleep duration is associated with higher incidence of periodontitis. This result did not correspond to general knowledge that the relationship between sleep duration and morbidity shows a U-shaped pattern. We assumed that the differences between our findings and these previously reported results occurred because they used a periodontitis severity score that was calculated based on examination by public health dentists, whereas we used a 5-point subjective score assessment to evaluate oral health status. This was one of the limitations of our study, as the definition of oral health status is relatively subjective. However, a condition like burning mouth syndrome, which is common and closely related to oral health status in the elderly, can be defined by subjective oral pain without any abnormal findings in the mouth.^[32]^ It has been demonstrated that both subjective symptoms and objective findings of the mouth should be taken into consideration when evaluating oral health status.^[33]^ Moreover, a Finnish nationwide comprehensive health survey suggested that self-assessed oral health could be used for screening purposes for oral health service planning and for priority allocation in large adult populations.^[34]^ In this regard, subjective score of oral health could be a more appropriate tool than objective examination by clinicians when assessing general oral health status. In addition, self-rated oral health is a valid and useful factor of overall oral health status and quality of life.^[35]^ Self-rated oral health has been assessed frequently in epidemiological studies, including national health surveys, and is a valid and useful means of screening overall oral health status.^[36,37]^

Sleep duration may be abnormally short or long, although the definition of proper sleep duration varies between studies, nations, cultures, individuals, etc. A sleep duration of 7–8 hours is recommended to the public for a general healthy lifestyle in Korea.^[18]^ Therefore, short sleep duration has usually been defined as <6 hours on average and long sleep as ≥9 hours. Both short and long sleepers are more likely to be in poorer overall health and to have been diagnosed with more medical conditions than normal sleepers.^[14,15,17]^ Appropriate sleep is important in terms of both general health and quality of life.

This study showed that short or long sleep duration is associated with poor oral health status. There are several possible explanations for the detrimental effects of short or long sleep duration on oral health status. First, the immune system impairment due to long or short sleep duration may increase susceptibility to periodontal disease, which involves the interaction of bacterial, host, and environmental factors.^[38]^ Second, adequate sleep is important for the maintenance of normal mental and physical function. When an individual feels stressed, adrenaline and stress hormones (e.g., cortisol) are released to prepare the body for the “fight-or-flight” response.^[36]^ Cortisol also acts to suppress the immune system, allowing bacteria to flourish in the mouth, and produces proinflammatory cytokines.^[37]^ Such an overload of bacteria in the mouth and changes of cytokine can lead to poor oral health, which may constitute a basis for the possible association between poor oral health and short or long sleep duration. Finally, short or long sleep duration has been shown to be part of an unhealthy lifestyle, which in turn may impair oral health.^[39]^ The quality of sleep may be worsened, and the excessive or deficient sleep duration may exacerbate oral health status. However, as this study did not assess sleep quality, we cannot reach any conclusions regarding these effects.

In this study, the effects of sleep duration on oral health were greater in younger people under 60 years old than those over 60 years old. Subjects younger than 60 years who slept for ≤5 h/day and ≥9 h/day had 1.4-fold and 1.3-fold higher prevalence rates of poor oral health status. However, sleep duration was not significantly associated with oral health status in the group over 60 years. We assumed that sleep duration had already decreased due to the aging process and that the circadian rhythm had been disrupted in the group over 60 years old.^[40]^ In fact, older people have more co-morbidities that influence general health than younger people. The causes of poor oral health in older people are multifactorial. In particular, saliva secretion affects oral health. In the group over 60 years old, the decrease of saliva secretion as a process of aging would be more likely to affect oral health than sleep.^[41]^

There was a sex-related difference in the relationship between short or long sleep duration and oral health status in this study. That is, excessive sleep duration exacerbated oral health status in men, while excessive sleep duration was not associated with poor oral health in women. Oral symptoms, such as xerostomia and burning mouth syndrome, have been shown to increase in association with menopause .^[42]^ Therefore, we hypothesized that hormones, such as estrogen, may play more important roles than sleep duration in the development of poor oral health. In addition, women are known to have poorer sleep quality and higher risk of insomnia than men because of the effects of the menstrual cycle and hormones.^[43]^ Therefore, long sleep duration may improve sleep quality or insomnia in women rather than in men, and this may be interpreted as having a positive impact on oral health.

Our study had some limitations. First, we did not differentiate oral health status according to types or causes. KNHANES contains some dental records such as the number of dental caries or prostheses, the color of teeth, and the number of tooth loss. The use of dental records to assess oral health might lead to a more detailed analysis. However, the comprehensive oral health status includes not only dental status but also oral mucosal status, limitation of chewing. Besides, a study showed that subjective oral health questionnaires are highly related to objective oral cavity status.^[35,44]^ Second, the cross-sectional nature of our study did not allow us to confirm a causal effect of sleep duration on the prevalence of poor oral health status. Therefore, it could not be determined, which is the cause and which is the effect with regard to short or long sleep duration and poor oral health. Nevertheless, this study had several strengths. This was the first study to evaluate the relationship between sleep duration and oral health status in the Korean population. Second, we used data from the KNHANES, a representative sample of the Korean population, and adjusted for many variables that influence the relationship between sleep duration and poor oral health. Therefore, we were able to demonstrate an association between poor oral health and short or long sleep duration.

## 5 Conclusions

In this national population-based cross-sectional study, we found that both short and long sleep durations were significantly associated with poor oral health. The association between short or long sleep duration and poor oral health status was more marked in men than in women. Short or long sleep duration may affect oral health status in adults under 60 years of age but not in those over 60 years old. Further longitudinal studies are required to define the association between sleep duration and oral health status.

## Acknowledgments

We thank the 150 residents of the otorhinolaryngology departments of the 47 training hospitals in South Korea and the members of the Division of Chronic Disease Surveillance in the Korea Centers for Disease Control & Prevention for participating in this survey and for the dedicated work they provided.

## Author contributions

**Conceptualization:** Jung-Hae Cho.

**Data curation:** Donghyun Jee.

**Investigation:** Jung-Hae Cho.

**Methodology:** Jung-Hae Cho.

**Software:** Donghyun Jee.

**Supervision:** Donghyun Jee, Yun-Jin Kang, Yong-Jin Park.

**Writing – original draft:** Sungjun Han.

**Writing – review & editing:** Jung-Hae Cho.
